# Analysis of Jersey versus Holstein breed profitability on north central US dairies

**DOI:** 10.3168/jdsc.2023-0371

**Published:** 2023-07-21

**Authors:** Lynn A. Olthof, Joseph J. Domecq, Barry J. Bradford

**Affiliations:** Department of Animal Science, Michigan State University, East Lansing 48824

## Abstract

With over 9 million cows in the United States, Holstein is the dominant breed in the US dairy population; however, the US Jersey population is growing. The objective of this study was to determine the profitability of Holstein and Jersey cows managed similarly on the same farms. Holstein and Jersey economic performance was compared within 3 north central US dairies, each milking more than 500 cows. The herds' average distribution was 21% Jersey (27 ± 0.67 kg/d milk, 4.92% ± 0.24 fat, 3.72% ± 0.03 protein) and 79% Holstein (37 ± 1.98 kg/d milk, 3.85% ± 0.21 fat, 3.17% ± 0.17 protein). A comparative budget approach was used to assess economic factors that differed between the breeds on a per cow annual basis, based on the assumption that an existing farm would be constrained by stalls and parlor to an equal number of Jersey and Holstein cows. Data from 2020 were gathered from farm management software, on-farm evaluations, and producer interviews. Sensitivity analysis was performed to determine which conditions would lead to different conclusions. Factors considered in the analysis included milk and component production, milk bonuses, ration prices, and dry matter (DM) intake. In a 2021 price scenario, Holstein cows ranged from $345 to $601 more profitable than Jersey cows on a per cow annual basis. Although Jersey cows had an advantage in component concentration, Holstein cows produced 13 ± 4.7% more fat and 22 ± 6.6% more protein annually due to greater milk yield. This accounted for most of the profitability advantage for Holsteins; 78% of the revenue advantage for Holstein cows came from increased component production. Few health and reproductive differences were found. The sensitivity analysis revealed, if all other factors remained the same, Jersey profitability would equal that of Holstein if any of the following changes occurred (assuming no change in Holstein metrics): mean Jersey milk production increased to 31 kg/d; milk price adjustments decreased from −$0.008 to −$0.11 per kg fluid milk; lactating cow ration price increased from $0.27 per kg DM to $0.53 per kg DM; or Jersey DM intake decreased from 20 to 15 kg/d. The study did not consider crossbred profitability or new infrastructure investments. In conclusion, Holstein cows were more profitable than Jersey cows on these 3 north central US dairies.

## Highlights

• Holstein cows are on average $456 more profitable than Jersey cows per cow annually.
• Most (78%) of the breed difference in revenue comes from milk component sales.
• Jersey cows are more feed efficient.
• The most plausible change in outcomes is a 10% improvement in Jersey productivity.

## Graphical Abstract

**Figure:**
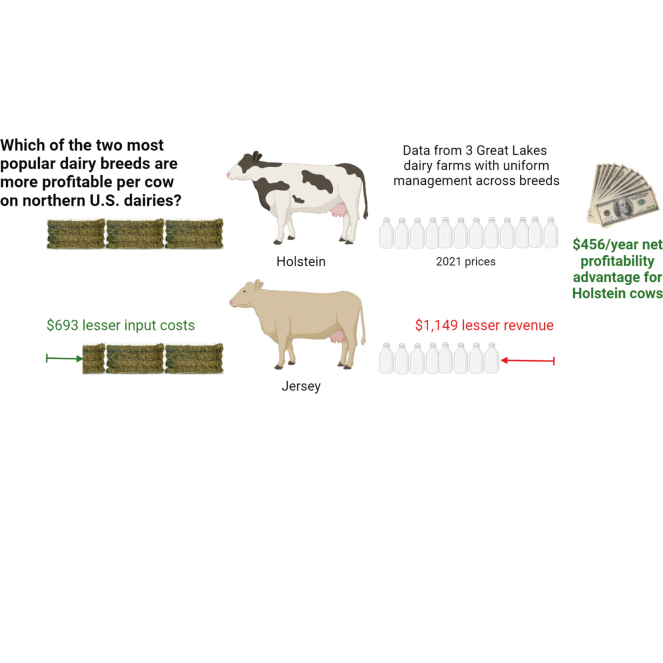
**Summary:** The 2 most popular dairy breeds in the United States are Holstein and Jersey. These 2 breeds bring different attributes to dairies, leading us to investigate which breed is more profitable. Data from 3 dairy farms managing both Jersey and Holstein cows were collected and analyzed using comparative budgets. Additionally, sensitivity analysis was performed to identify factors with the potential to change profitability conclusions. Evaluating the breeds in a north central US context, on the same farms with the same management, Holstein cows were more profitable than Jersey cows on each of the 3 farms, with an average advantage of $456 per cow annually.

Dairy farms continue to strive for ways to improve profitability and thereby sustain their business. The question of whether Jersey or Holstein cows are more profitable has remained relevant, particularly as producers face increasing feed input costs and volatile milk prices (Endres, 2018). According to the Council on Dairy Cattle Breeding, in 2000, Holstein cows made up 92.3% of the US dairy herd and Jersey cows made up only 3.8%. In 2020, Holstein made up 79.9% and Jersey made up 7.9% of the US dairy herd, with 11.8% constituted by crossbred animals (USDA, 2000; CDCB, 2020). Although Holstein clearly remains the dominant dairy breed in the United States, the Jersey breed has a growing population, particularly in the Southwest region (Garcia-Peniche et al., 2005).

In evaluating the economics of breed selection, the milk pricing system can influence the outcome (Schmidt and Pritchard, 1988). Most US dairy producers are paid for component yields rather than fluid milk; therefore, economic analyses need to focus on yields of fat, protein, and solids nonfat and protein (**SNFP**) rather than fluid yield or component concentrations alone. On a fluid milk basis, Holstein cows were reported to produce 23% more milk than Jersey cows (White et al., 2002). Jersey cows produce milk with greater fat and protein concentrations, but these do not fully overcome the lesser milk yield, resulting in lesser component yields for Jersey cows (Palladino et al., 2010). On the cost side, Jersey cows consume less feed than Holstein cows (Beecher et al., 2014), and reproductive differences may also exist. In an analysis of over 5 million cows, Jersey cows had 1% to 11% greater conception rates than Holstein cows (Norman et al., 2009). These lesser costs could allow Jersey cows to match Holstein profitability despite lesser milk component yields.

Previous research comparing Jersey and Holstein cows often contrasted the breeds using different farms with different management and environments (Bailey et al., 2005; Norman et al., 2009; Xue et al., 2011; Kristensen et al., 2015; Lim et al., 2020). Research also has not accounted for bonuses and discounts that are paid on a fluid milk basis (Bailey et al., 2005) or for potential differences in health outcomes. The objectives of this study were to identify whether Jersey or Holstein cows are more profitable in existing North Central region dairy facilities and to determine which conditions might influence this conclusion.

Commercial dairy farms were recruited for participation using the following criteria: measurement of individual cow milk yields and component concentrations at least 8 times per year; at least 5% of the farm's herd and one pen representing each breed; both breeds located on the same farm but generally housed in separate pens; and both breeds comprised of mature populations with a stable parity distribution. Neither Institutional Animal Care and Use Committee nor Institutional Review Board approval were required for this study because no changes in animal management were introduced and humans were not the subject of data acquisition. Three North Central region dairies were identified for the study. All 3 farms provide freestall housing with sand bedding and concrete floors, and milk 2 or 3 times per day. Animals on the same farm had the same management and environment unless otherwise noted. Table 1 provides an overview of the characteristics of the 3 farms. On average, the study herds comprised 21% Jersey and 79% Holstein cows. All 3 farms added Jersey cows to their herds over the past decade for a variety of reasons, including adding revenue from dairy sales and increasing milk components shipped within a limited volume quota.

**Table 1:** Characteristics of the 3 dairy farms used to evaluate profitability of Holstein and Jersey cows

Item^1^	Farm 1		Farm 2		Farm 3	
	Holstein	Jersey	Holstein	Jersey	Holstein	Jersey
Number of cows	867	448	3,035	189	651	208
Percent of herd	66	34	94	6	76	24
Fat (%)	3.75	4.90	4.10	4.70	3.71	5.17
Protein (%)	3.12	3.74	3.36	3.69	3.03	3.73
Fluid milk yield (kg/yr)	13,489	9,782	11,684	8,777	13,232	8,376
Fat yield (kg/yr)	514	473	487	413	491	433
Protein yield (kg/yr)	424	367	399	324	401	312
SNFP yield (kg/yr)	757	529	666	475	742	453
Total component yield (kg/yr)	1,695	1,370	1,551	1,211	1,635	1,199
Turnover rate (%)	27.9	33.4	42.4	40.0	38.7	37.5
Mean DIM (d)	157	177	200	190	168	147
Lactating DMI (kg/d)	26.3	21.8	25.7	19.7	25.9	20.0
Calving interval (mo)	13.6	13.5	13.0	12.9	13.4	12.8
21-d pregnancy rate (%)	29.2	26.7	22.9	23.0	31.6	43.0
Pregnancies per AI (%)	34.1	30.6	42.3	40.0	28.1	35.7

1 SNFP = solids nonfat and protein.

Data were collected through farm visits, herd management software, and conversations with the producer to understand the producer's goals. Understanding the goals of each operation created awareness of unique farm circumstances that influenced management decisions and data interpretation. For example, one farm sold a substantial number of lactating cows to other herds and these sales needed to be accounted for to calculate an unbiased herd turnover rate. We used 2020 data for milk production, reproduction, health, and other cow performance records, as that was the last full year of records when the study began. We identified some key data gaps on each farm, for which values were estimated during the analysis. Farms 1 and 3 did not have accurate DMI measures for either breed. Farms 2 and 3 did not have calf health records and farm 3 housed Holstein cows in a newer, better-ventilated barn compared with Jersey cows.

From PCDart (Dairy Records Management Systems) and DairyComp305 (Valley Ag Software) herd management software, we determined the average annual milk, fat, and protein yields for each parity (1, 2, and 3+) by breed. Lactose content of 4.72% was used for Jersey cows and 4.85% for Holstein cows to calculate the SNFP sales (Lim et al., 2020). For farms 1 and 3, we used the formula of the National Academies of Sciences, Engineering, and Medicine (NASEM, 2021) to estimate DMI for each breed, whereas recorded DMI data were used for farm 2. Several factors affected the DMI model; we used a parity factor of 0.67 to account for the age distribution in both herds, an average BCS of 3, and estimated mature cow weights of 544 and 681 kg for Jersey and Holstein cows, respectively. Using the statistics from Table 1 to populate the equations, for farm 1 we computed a predicted DMI for Jersey cows of 21.8 kg/d and 26.3 kg/d for Holstein cows. For farm 3, the predicted DMI was 20.0 kg/d for Jersey cows and 25.9 kg/d for Holstein cows. The same formula was used to estimate dry cow DMI with the milk energy at 0 and BCS at 3.75. With these parameters, for farm 1, Jersey dry cows were estimated to consume 13.7 kg DM/d and Holstein dry cows 16.7 kg DM/d. The NASEM (2021) equation predicted farm 3 Jersey dry cows to consume 12.7 kg DM/d and Holstein dry cows 16.7 kg DM/d.

We also compared reproductive statistics and costs between breeds. On each farm, if Jersey and Holstein conception rates differed by less than 4% and services per conception differed by less than 0.5 services between breeds, reproductive efficiency was considered not sufficiently different to be included in the analysis. This decision was based in part on the uncertainty around these estimates and in part on the relative impact of small magnitude differences on overall profitability. Farm 3 was the only dairy determined to have different reproductive performance between the breeds. Farm 3 also used a heat detection (activity) system on Holstein cows but not Jersey cows. Reproduction was accounted for by adding the yearly per cow cost for the activity monitoring system to the cost of a conception per Holstein cow. The cost per conception was calculated by multiplying services per conception by cost per service for each breed. Although reproduction costs were not included in the comparative budgets of farms 1 or 2, minor reproductive differences influenced calving interval, milk yield (affected by average DIM), and age at first calving, which were factors in comparative budget calculations. Turnover rate is affected by many factors including reproduction, and is not directly reflective of reproductive performance.

Cow and calf health were assessed separately. All 3 farms recorded milk fever, retained placenta, metritis, respiratory, ketosis, displaced abomasum, and mastitis cases, which were used to calculate total annual disease costs for each breed. Although case definitions were not uniform across dairies, uniform management within herds provides confidence for the disease incidence comparisons across breeds within herd. All health data were calculated on an annual risk basis (annual cases divided by steady-state number of cows, by breed) and multiplied by the disease's respective treatment cost (APHIS USDA, 2013; Liang et al., 2017). We had limited information for calf health and were only able to include calf health records for farm 1, including pneumonia and scours cases. The risk of pneumonia or scours was calculated by breed (annual cases divided by the total number of calves raised in that year). The costs associated with each disease were then used to calculate the total cost on a risk of a case for pneumonia and scours for Jersey and Holstein calves (Schneider et al., 2009; Mohd Nor et al., 2012). On this farm, 70 pneumonia and 44 scours cases were recorded for calves with no breed designation. Therefore, we used the breed proportion of the assigned cases of pneumonia and scours to distribute the unassigned events to breeds, providing a more representative total disease cost for the calves. Due to the lack of calf mortality records, we were not able to factor mortality into the analysis, but the producer-indicated mortality rates were similar between breeds. Without calf health data on farms 2 and 3, we could not assess differences between breeds in calf loss or health costs for these farms.

Bringing all economic factors together, a comparative budget was constructed on a per cow annual basis for each dairy. The revenues included protein, fat, and SNFP sales, cull cow sales, value of calves born, and milk bonuses. Expenses included milk transport, milk discounts, feed, manure handling, heifer raising, cow health, calf health, and reproduction costs. Labor was only included in reproduction and disease treatment costs, as other labor costs were assumed to be the same per cow across breeds. Bonuses for low SCC and other milk bonuses and charges were applied on a fluid milk basis, whereas component sales were applied on a solids basis (per practices of milk cooperatives). Heifer raising was factored into the analysis on a risk of leaving the herd basis; for example, if a breed within a herd had a 30% turnover rate, cows in that breed were charged 30% of the cost of raising a heifer from birth to first calving. Cow and calf health costs were valued based on risk of cases annually. Costs or benefits that were not apparently different between the 2 breeds were not included within the comparative budget, as already mentioned for reproduction.

Due to the impact of the COVID-19 pandemic on the dairy markets in the United States in 2020, we used average 2021 prices for milk components, milk bonuses, feedstuffs, and animals, as it was a more representative year for dairy markets. We used average prices for the year 2021 from the Mideast Milk Marketing Order of $4.168/kg for fat, $6.091/kg for protein, and $0.852/kg for other solids to calculate milk value (USDA, 2022). Two different milk cooperatives were represented within the study, and fluid milk bonuses and charges were determined from producer milk checks. A standard SCC bonus structure was used across all herds, with a bonus (all per kg fluid milk) of $0.004 for SCC from 180,000 to 200,000; $0.009 for SCC from 170,000 to 180,000; $0.013 for SCC from 160,000 to 170,000; $0.018 for SCC for 150,000 to 160,000; and $0.022 for SCC under 150,000. For the producer price differential, we used −$0.0013 per kg of fluid milk, which was the 2021 average from the Mideast Milk Marketing Order (USDA, 2021). A producer transport cost of $0.014 per kg was used for all farms. Bonuses, charges, and discounts applied on a fluid milk basis were collectively referred to as milk price adjustments. We valued an AI service at $39.60, which included farm costs of semen, labor, and synchronization program hormones, which came from the producers.

Farm-level feed ingredient costs were used in feed cost calculations. A standard value for calves and cull cows was used across farms based on market prices; Jersey and Holstein cull cows were valued at $450 and $750, respectively, whereas Jersey heifer and bull calves were valued at $100 and $25, respectively, and Holstein heifer and bull calves were valued at $150 and $125, respectively. Disease costs accounted for veterinary, treatment, discarded milk, and labor costs, as it was assumed that decreased milk production, culling, reproduction, and death were already accounted for within our comparative budget. Regardless of breed, the cost per case of milk fever was $97.84, retained placenta was $96.63, metritis was $140.92, respiratory disease was $28.60, ketosis was $64.20, displaced abomasum was $212.72, and mastitis was $154.71 (APHIS USDA, 2013; Liang et al., 2017). Disease costs per case were then multiplied by the risk of a case for each condition to determine annualized disease costs per cow for each breed. Calf scours and pneumonia treatment and labor were priced at $11.00 and $38.00 per case, respectively (Schneider et al., 2009; Mohd Nor et al., 2012).

Results from comparative budgets revealed that Holstein cows are more profitable than Jersey cows on these 3 north central US dairies (Table 2). Within the comparative budget, component sales accounted for between $708 and $1,029 (or 72 to 86%) of the total revenue difference between the breeds, with Holstein cows producing an average of 367 ± 60 (SD) kg more milk solids than Jersey cows. After accounting for animal sales and fluid milk bonuses, Holstein cows generated an estimated $940 to $1,424 more total revenue than Jersey cows.

**Table 2:** Summary of the 3 farm comparative budgets with averages and SD for the study^1^

Revenue factor	Revenue change ($)			Mean	SD	Expense factor	Expense change ($)			Mean	SD
	Farm 1	Farm 2	Farm 3				Farm 1	Farm 2	Farm 3		
Protein sales	(345)	(458)	(542)	(448)	99	Milk transport	(51)	(69)	(108)	(76)	29
Fat sales	(170)	(310)	(241)	(240)	70	Feed costs	(415)	(431)	(478)	(441)	33
SNFP sales^2^	(194)	(163)	(247)	(201)	42	Manure handling	(40)	(34)	(54)	(43)	10
Cull cow sales	(60)	(138)	(122)	(106)	41	Heifer raising	(3)	(203)	(100)	(102)	100
Calf value	(66)	(66)	(70)	(67)	3	Cow health	(10)	(4)	(20)	(11)	8
SCC bonus	(57)	92	(140)	(35)	117	Calf health	2	—	—	1	1
Milk price adjustments	(49)	(40)	(64)	(51)	12	Reproduction	—	—	(64)	(21)	37
Total revenue change	(940)	(1,084)	(1,424)	(1,149)	249	Total expense change	(517)	(740)	(823)	(693)	158
Net change in profit (switching from Holstein to Jersey)							(422)	(345)	(601)	(456)	131

1 The comparative budget was determined on a per cow annual basis. Changes were calculated through subtracting Holstein figures from Jersey figures.

2 SNFP = solids nonfat and protein.

In total, Jersey cows had annual expenses of $517 to $740 less than Holstein cows. All expense categories showed an advantage for the Jersey breed. We hypothesized that the Jersey breed would have an advantage in heifer raising costs due to their smaller size and lesser feed requirements (Beecher et al., 2014). We indeed found that the total variable costs (excluding infrastructure) to raise a Jersey heifer ranged from $1,275 to $1,379, whereas Holstein heifer rearing costs ranged from $1,521 to $1,681. On farm 1, individual calf milk consumption data were available due to use of robotic calf feeders. We did not have starter intake data but utilized research from Terré et al. (2007) to estimate Holstein calf starter intake at 19.48 kg over the preweaning period. Milk intake data showed that Jersey calves consumed 80% of the milk that Holstein calves consumed, and this proportion was applied to predict starter intake for the Jersey calves (15.58 kg). On all 3 dairies, Jersey heifers were older at first calving than the Holstein heifers, diminishing the cost advantages of raising Jersey heifers. This could be partially attributed to producers noting more Jersey calf health events, potentially disrupting growth. Furthermore, heifer raising costs were factored into the comparative budget based on the number of heifers needed to replace cows leaving the herd. Farm 1 had an annual turnover rate of 33.3% for Jersey cows and 27.9% for Holstein cows, resulting in annual costs of raising replacement heifers for a Jersey cow of $459 compared with $462 for a Holstein cow, giving just a $3 advantage to Jersey cows on this farm. Across farms, annual Jersey replacement costs ranged from −$203 to −$3 relative to Holstein.

The total profitability difference revealed that the reduced expenses for Jersey cows did not compensate for the revenue lost compared with Holstein cows. The net change in profitability for switching from Holstein to Jersey cows on these farms ranged from −$345 to −$601 per cow annually, a substantial net loss. To put this in context, a farm financial database including 414 dairies primarily in the north central United States reported a median net profit of $18.85/cow for 2021 (FINBIN, 2023), meaning that the loss in profitability for a Jersey versus Holstein cow would dwarf the small profit margin for a typical farm that year.

Before the study, we anticipated an advantage for Jersey cows in fluid milk bonuses and charges. On farm 1, Jersey cows had more consistent and lower SCC throughout the year but had a larger advantage in the summer. With this significant SCC gap in summer—consistent with reported heat stress resilience of the Jersey breed (Smith et al., 2013)—Jersey cows were able to capture an additional $0.004/kg bonus in June and August and an additional $0.013/kg in July compared with Holstein cows. However, because SCC bonuses are paid on a fluid milk basis, the net SCC bonus revenue was still greater for Holstein than Jersey cows in these months (due to greater fluid yield). This same seasonal SCC pattern was observed on farm 2 but not on farm 3, where lactating Jersey cows were housed in an older, less ventilated barn.

Sensitivity analysis was used to investigate factors that could potentially alter profitability conclusions. Jersey daily milk production, milk bonuses and discounts, and Jersey DMI were varied for this analysis, with all other factors held constant (Figure 1). The sensitivity analysis revealed that Jersey profitability would equal that of Holstein (assuming no changes in Holsteins) if any of the following changes occurred: Jersey milk production increased to between 29.5 and 32.6 kg/d (79%–87% of Holstein production); milk price adjustments decreased from −$0.008/kg fluid milk to between −$0.10 and −$0.12 per kg fluid milk; lactating cow TMR price increased from $0.23 to $0.29/kg DM to between $0.40 and $0.59/kg DM; Jersey DMI decreased from between 19 and 21 kg/d to between 13 and 17 kg/d; or the DMI NASEM (2021) formula overpredicted Jersey DMI by 20% to 34%. Although additional DMI would be required for increased Jersey milk production, this is accounted for in the productivity sensitivity analysis. The sensitivity analysis strengthened the conclusion that Holstein cows are more profitable than Jersey cows on these 3 north central US dairy farms; extreme changes would have to take place for Jersey cows to be more profitable on these farms.

**Figure 1:**
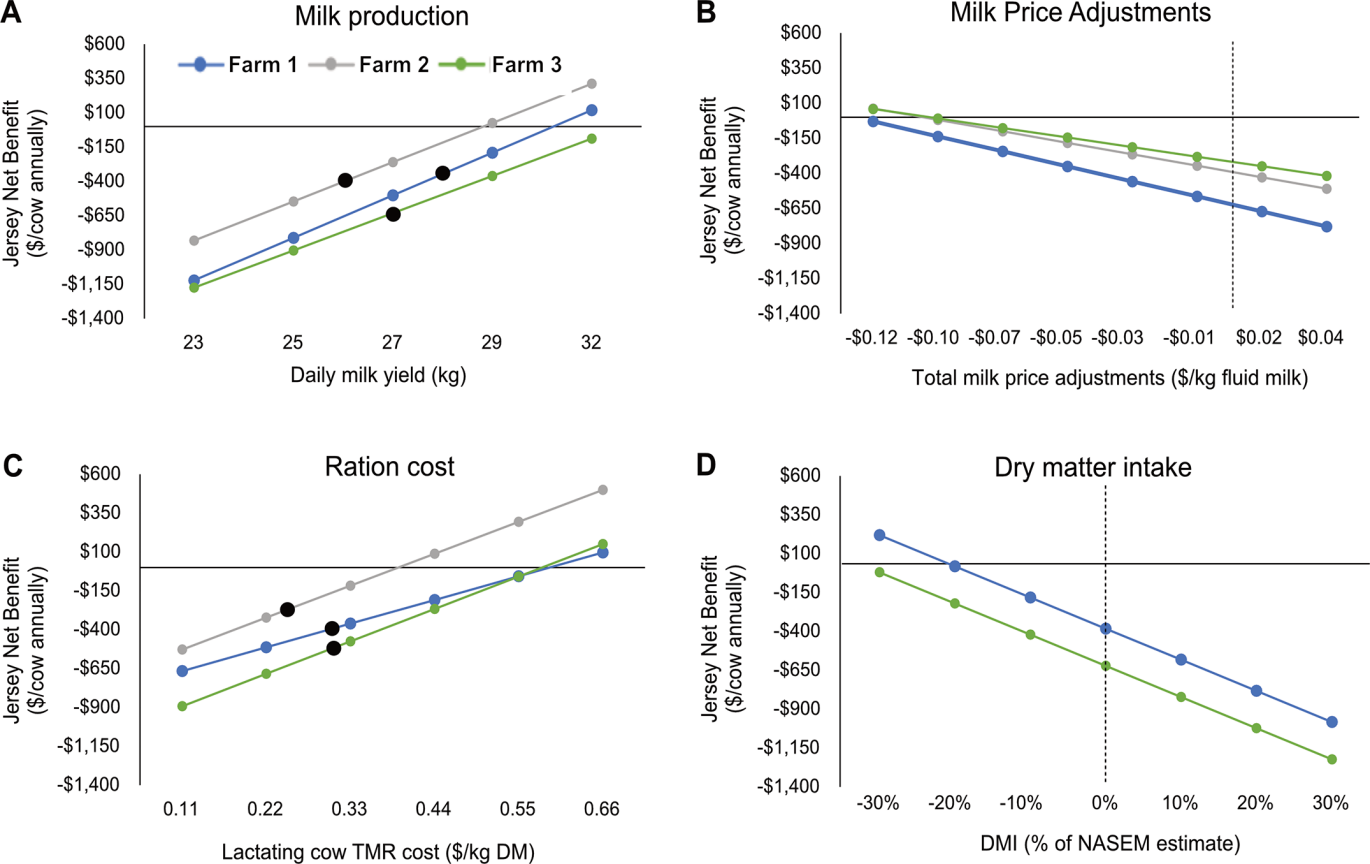
Sensitivity of profitability results to changes in key input variables. Each panel shows the net profitability advantage of replacing a Holstein cow with a Jersey cow in response to varying (A) Jersey daily milk production, (B) total milk price adjustments, (C) lactating cow TMR price, or (D) Jersey DMI relative to NASEM (2021) DMI model estimates for the 2 farms without DMI data. For each sensitivity analysis, all other factors were held constant, with the exception that DMI was adjusted to align with increasing milk production for panel A. The black dots and dashed lines represent the scenarios evaluated in the study.

The key takeaways are that greater fat and protein yields—despite lesser concentrations—put Holstein cows at a significant profitability advantage over Jersey cows. Because milk bonuses on these farms were greater than the discounts and charges applied on a fluid basis, the greater volume produced by Holstein cows added to their advantage. Although the differential between Jersey and Holstein cows varies with market conditions (Figure 1), greater Jersey productivity was the only factor considered in the sensitivity analysis that could plausibly change the outcome with current market structures.

There are some caveats in how these findings should be interpreted. First, these conclusions apply to a north central US climate and pricing environment. We also based the analysis on use of existing facilities; building new facilities may change our conclusions, as a given infrastructure investment could house more Jersey cows. The conclusions may be influenced by unique revenue streams on some farms (e.g., breeding animal sales). Last, we did not have data available to assess crossbred performance within herds.
